# Transferring patients’s experiences of change from the context of physiotherapy to daily life

**DOI:** 10.1080/17482631.2020.1735767

**Published:** 2020-03-09

**Authors:** Tove Dragesund, Aud Marie Øien

**Affiliations:** Faculty of Health- and Social Sciences, Western Norway University of Applied Science, Bergen, Norway

**Keywords:** Awareness, musculoskeletal pain, focus groups, physical therapy

## Abstract

**Purpose**: In the treatment of patients with long-lasting musculoskeletal pain, the challenge is to identify causal and sustaining factors and targeted treatment in order to improve function. Norwegian Psychomotor Physiotherapy (NPMP) is an approach often applied to patients with such pain. Long-term NPMP processes from the patients’ perspective have been studied and discussed in the light of phenomenology of the body.

The study purpose was to explore what kind of changes patients with long-lasting musculoskeletal pain experience during NPMP and further transfer into daily life context.

**Methods**: A phenomenological, descriptive, and retrospective design was applied. Two focus-group interviews were conducted with 11 patients receiving such treatment. The interviews were audiotaped, transcribed, and analysed inspired by Giorgi’s phenomenological methodology.

**Results**: The analysis resulted in an overarching structure: “To develop embodied ownership of oneself over time”, and two themes describing the essence of change that the patients experienced: (1) “To get an embodied grip on oneself through treatment”; (2) “To give oneself space in daily life”.

**Conclusions**: Enhanced embodied self-perception involving a sense of embodied ownership and agency seemed to be important both to be aware of own bodily needs and to transfer changes from treatment into daily life.

## Introduction

Long-lasting musculoskeletal pain is a complex phenomenon with a multifactorial aetiology, including biological, psychological, and social factors (Malterud, 2000; Steihaug, 2005). In western countries, doctors often regard such pain as incomprehensible characterized by pronounced symptoms despite normal findings on clinical, blood, and radiologic tests (Malterud, 2000; Steihaug, 2005). During the examination of patients with such pain problems, the challenge is to identify causal and sustaining factors as well as targeted treatment (Malterud, 2000; Steihaug, 2005). Physiotherapists are often treating patients with long-lasting musculoskeletal pain. During their examination of these patients, they might detect bodily signs linked to the pain problems, like changes in posture and balance, restricted breathing, and increased muscle tension (Kvåle, Ljunggren, & Johnsen, 2003a). These elements are also addressed in the further treatment.

The phenomenological perspective of the body might be useful in improving the understanding of long-lasting musculoskeletal pain and how to target treatment of patients with such pain problems (Gallagher & Zahavi, 2012; Leder, 1990; Merleau-Ponty, 2012). From the perspective of phenomenology of perception, Merleau-Ponty (Gallagher & Zahavi, 2012) claims that the body is the foundation of experience and the basis from which we perceive and interact. Thus, we are situated in the world. This perspective is decisive but does not mean that the person takes precedence over the world, rather that the person and world are inseparable (Gallagher & Zahavi, 2012). The body is defined as subject and first person and from which any understanding of human experience begins (Merleau-Ponty, 2012). Further, the perspective outlines the body’s ambiguity as something the person is and at the same time has and that lived life becomes incorporated in the body (Merleau-Ponty, 2012). As such, experiences in life make an imprint in and on the body.

Perception is contextual and based on the relation between the figure and the background (Gallagher & Zahavi, 2012). This phenomenon implies that one may shift the dimensions of the body into the background of one’s attention or allow them to be an object of one’s attention. This may indicate a shift from a pre-reflexive towards a reflexive awareness of specific bodily reactions.

Habits are incorporated practices, always related to meaning. We express ourselves through habits, interactions, and coexistence with the world and others without necessarily being aware of it (Gallagher & Zahavi, 2012).

Phenomenological philosophy may provide basic assumptions supporting the theoretical perspective of Norwegian Psychomotor Physiotherapy (NPMP). The treatment approach is often applied to patients with long-lasting musculoskeletal pain and is the context of the present study. The theoretical perspective of NPMP is that physical, psychological, and social strain influence the body as a functional unity affecting muscle tension, respiration, posture, balance, and movements. Muscle pain might be caused by sustained muscle contraction suppressing unpleasant emotions (Braatøy, 1947). Accordingly, the whole body in contrast to parts of the patient’s body is addressed during the first encounter and the following treatments (Thornquist & Bunkan, 1991). NPMP is individualized and process oriented. The overall aim of the approach is to readjust posture and harmonize muscle tension, breath, and movements by means of bodily interaction consisting of specific guiding of movements by touch, i.e., “hands-on” and massage, as well as verbal interaction. The approach also includes helping the patients to recognize and change habitual muscular tensions involved in the regulation and inhibition of emotional experiences (Braatøy, 1947). Awareness of own bodily reactions, like patterns of tension and movements, is therefore considered as an important prerequisite for functional change, which may also include emotional change (Thornquist & Bunkan, 1991). As part of the treatment process, an improved sensation of the body might follow. This opens the possibility of becoming more in touch with one’s own body and be able to regulate oneself during daily life activity (Dragesund & Raheim, 2008; Øien, Råheim, Iversen, & Steihaug, 2009; Sviland, Raheim, & Martinsen, 2012).

Long-term NPMP processes from the perspective of patients with long-lasting musculoskeletal pain have been studied (Dragesund & Raheim, 2008; Ekerholt & Bergland, 2006, 2008; Øien, Iversen, & Stensland, 2007; Øien et al., 2009; Sviland et al., 2012). After completed NPMP, Ekerholt and Bergland (2006, 2008) explored the patients’ experiences of the massage and own breathing. They found that the massage enhanced relaxation as well as perception and reflection on own body (Ekerholt & Bergland, 2006). The experiences of breathing enabled the patients to understand the interaction between the rhythm of breathing and well-being. Awareness of own rhythm of breathing also seemed to give better access to own movements and emotional patterns (Ekerholt & Bergland, 2008). Øien et al. (2007, 2009) explored patients’ perception of bodily changes during NPMP. The experiences of change described by the patients were based on the reflection connected to the integrated hands-on and verbal approach in the treatment (Øien et al., 2007). The process of self-perception appeared to be a basis for reducing pain as well as being integrated with changing patterns of movement, breath, and expression, both within and outside therapy (Øien et al., 2009). The patients included in the above-mentioned studies have described factors like being more in touch with and familiar with own body and self, to be better acquainted with bodily reactions, to better interpret bodily symptoms, and to connect these reactions to relational dimensions and habitual ways of acting (Dragesund & Raheim, 2008; Ekerholt & Bergland, 2006, 2008; Øien et al., 2007, 2009; Sviland et al., 2012).

To summarize, few studies of NPMP have focused on which experiences of changes patients transfer from treatment into daily life. The purpose of the study was, therefore, to explore more in depth what changes patients with long-lasting musculoskeletal pain experience in treatment and how they transfer these experiences of change into their daily life context.

## Methods

In this study, a phenomenological, descriptive, and retrospective design was used to explore the patients' experiences of change from ended or still ongoing treatment and what they further had transferred into their daily life. Phenomenology is a systematic, philosophical approach describing the internal meaning structures of lifeworld experiences (Giorgi, 2009). The first-person perspective is thus crucial because experiences can only occur if they occur to someone. Thus, experiences should be examined in the way they occurred (Giorgi, 2009).

Phenomenology is also a methodological strategy to achieve and explore empirical data and to grasp new insight and a comprehensive understanding (Giorgi, 2009). In order to elucidate experiences from both within and outside the treatment, we wanted the reflections on lived experiences from the patient’s perspective. Reflecting on lived experience encompasses looking back in time, reflecting on experiences already lived through (van Manen, 2014). We searched a phenomenological attitude of openness to the patients’ experiences, allowing the phenomenon and its meanings to be displayed in unexpected ways (Giorgi, 2009). We tried to look at how the world shows up for the patients (Giorgi, 2009). This might imply making oneself available to how the phenomenon appears, by being sensitive and attentive to the experiences of others (Giorgi, 2009). We searched for such openness both to the research question and the patients’ experiences and to ourselves as researchers. The process is called bracketing, an ongoing active and sensitive attitude in the research situation.

Both authors are experienced NPMP specialists providing preunderstanding and scientific knowledge of the phenomenon. We have valuable insight concerning NPMP treatment processes because of clinical experiences for more than 35 years, as well as prejudices.

### Recruitment and participants

The patients recruited in the present study had received or were still receiving NPMP in an ongoing randomized controlled trial (RCT). As such, the present study is separate and not a part of the RCT. The inclusion and exclusion criteria for the patients were the same as in the RCT: (1) Being employed in the municipality; (2) Having several short sick leaves during the last 2 years, being sick-listed fulltime for <6 months, or working despite long-lasting widespread musculoskeletal pain or pain located to neck and shoulders. Exclusion criteria were (1) localized musculoskeletal pain (like knee, elbow, and hip pain); (2) sick-listed more than 6 months without interruptions of working.

Out of the 60 patients receiving NPMP in the RCT, we invited 25 to participate in this current study and 14 volunteered. However, three did not show up because of unknown reasons. Finally, 11 participated, 10 women and one man, aged from 34 to 67 years. They had received NPMP for 3–12 months, and nine had completed and two were still receiving the NPMP treatment. The patients’ pain problems were long-lasting widespread (*n* = 5), neck and shoulder (*n* = 4), and back pain (*n* = 2). Eight patients were working full time and three were partly on sick leave.

### Ethical considerations

The study was approved by the Western Regional Committee of Ethics in Medicine and registered at the Norwegian Social Science Data Service. The patients received written and verbal information about the aim of the study and stating that participation was voluntary. In order to protect the confidentiality, identifying information about the patients was removed during transcription.

### Focus-group interviews

In order to explore the patients’ experiences, the focus-group interview was chosen. Focus-group interviews are defined as collective conversations examining a particular set of relevant issues (Morgan, 1997). We conducted two focus-group interviews with different patients in each group. We included seven participants in the first interview and four in the second, one month later. The number of participants is in line with recommendations in the literature, which varies between 4 and 12 (Krueger & Casey, 2000; Morgan, 1997). The interview guide for the two interviews covered the broad themes: What kind of expectations for change did you have before the treatment? If any, what kind of changes did the treatment contribute? How do you sustain these achieved changes in daily life? We prepared follow-up questions about specific lived-through experiences to obtain rich descriptions, like: Can you give descriptions that are more detailed? Do you have some concrete examples?

In advance of the interviews, the first author and moderator noted own anticipations and thoughts about the phenomenon studied. The themes of the interview guide were sent to the participants, giving them the opportunity to reflect upon them before the interview. Both interviews took place in a quiet meeting room at the moderator’s workplace. They were audiotaped and lasted for two and a half hours. Initially, the moderator repeated the purpose of the study and encouraged the participants to describe their experiences from the treatment, giving each participant 10–20 min. Then, the patients commented and reflected on each other’s stories and experiences. Both interviews ended by summing up main points, providing for additional comments from the participants (Kvale & Interviews, 2009). The second author and co-moderator wrote field notes during each interview, describing her experience of the atmosphere of interaction among the participants. Both focus-group interviews gave rich descriptions and stories about the patients’ experiences according to the aim of the study. Accordingly, the sampling strategy was in line with the principles for qualitative studies (Polit, 2017). Immediately after each interview, the first author transcribed them verbatim.

### Analysis

In order to identify commonalities and differences in the patients' experiences, the analysis was inspired by Giorgi's (2009) phenomenological method. The aim was to reveal and describe the structure of the patients’ experiences at a more abstract level than the transcribed interviews. Both authors participated in the four different steps of the analysis. The first step started by reading the interviews and field notes, to grasp a global sense of the descriptions. In the second step, both authors established units of meaning and grouped them into categories related to the content. In the third step, “the heart of the method”, the units of meaning were further transformed into the researchers’ voice in line with Giorgi (2009). During this step, we considered and reconsidered the different aspects of the text to uncover and clarify the meaning of the patients’ experiences. In line with what Giorgi calls free imaginative variation, we tried to keep an open attitude and used our own experiences and knowledge (both being NPMP therapist) in order to understand, extract, and deepen the participants’ expressions in the text. We reflected on the meaning units while asking the question: What is this really about? In accordance withZahavi's (2018) recommendations, we also tried to grasp “how“ and “as what“ the experiences were presented, as well as to discover the intentional acts and experiential structures in relation to how the experiences might be understood. Finally and in the fourth step, the transformed units of meaning became the basis for searching for the general structure of the patients’ experiences (Giorgi, 2009).

## Results

The analysis resulted in an overarching general structure, which may be termed as an abstract, condensed description developed on the basis of the patients’ experiences from the treatment. We labelled this overarching structure “To develop embodied ownership of oneself over time”. Furthermore, the analysis crystallized two constituent subordinate themes that describe the essence of the change the patients experienced: “to get an embodied grip on oneself through treatment” and “to give oneself space in daily life”. These two themes are interwoven. We first present the overarching structure and then the two essential themes, with a brief summary of the depth and variations in the empirical data.

### To develop embodied ownership of oneself over time

All the patients described stories of physical and mental strain, both at work and at home. Before NPMP, their insight into how their lived lives and pain influenced each other varied. Some hypothesized that their complaints derived from physical strain only and wanted the therapist to remove the pain. Others experienced an impact of both physical and mental strain on their pain but did not know how to relate to this insight. During the NPMP treatment, all the patients gradually became more in touch with their bodily reactions and feelings, which included a greater ownership of themselves. Becoming more in touch with themselves in treatment seemed to increase their attention on bodily reactions and feelings, in daily life. These new experiences seemed to open up space to act differently in specific situations.

### To get an embodied grip on oneself through NPMP over time

Getting an embodied grip on oneself was a matter of how NPMP treatment over time contributed to an attention to and awareness of own body, movements, and patterns of action, such as tensing up due to imposed strain. This enhanced awareness opened access to feelings, including personal learning and development.

Prior to receiving NPMP treatment, several patients expected that the treatment would focus only on painful areas, with the use of different technological equipment. They had never heard about the treatment approach and were surprised, and some also sceptical, of the flexible approach of NPMP including conversations about bodily sensations (like restricted breath and tense muscles), hands-on related to massage, guiding and facilitating movements, and rest in order to reduce tension. One patient memorized the first encounter:

I have experienced physiotherapy as very physically oriented and imagined NPMP treatment being similar. I was unaware that my pain could relate to other factors than physical ones. In the first session, the physiotherapist explained that the pain could attribute to the strain I seemed to put on myself. She further explained that she could not promise me to be free of pain, but rather to be more aware of the strain I put on myself. This made me reconsider, if I should spend time on this treatment. Nevertheless, I decided to try to get something out of it.

Gradually, the patients experienced how they during the treatment became more in touch with their own bodies and feelings. This process varied in time among the patients. One patient, with a lot of strain related to a drug-addicted son, described the first part of the treatment like this:

I stress a lot, and do hard physical exercises because I think this will improve my pain condition. I never give in even if it hurts a lot. At the start of treatment, I was unsure about the purpose of the conversation, as well as why the therapist stopped when I expressed pain during the “hands on”. I wondered if the treatment would be effective. Gradually, however, I became calmer and in touch with my own repressed feelings, like sadness and grief.

Another patient expressed sadness and started crying as she recounted her treatment process. She had demanding experiences with a disabled son and suffered from sustained muscle stiffness and pain. She told:

Very much, my son’s needs determined my movements and daily routines. For years, I had to ignore my bodily signals of tiredness and pain. In the treatment, I enjoyed to become in touch with my own body again.

Most of the patients appreciated the flexible, gentle approach, continually adjusted to suit their personal needs. They experienced that the treatment alternated between conversation and “hands-on”. If they experienced periods of increased pain and discomfort, more “hands-on” was used. Time of conversation expanded if incidents of strain occurred. Recurrent topics explored during the treatment included daily movements such as sitting, lying, standing, and walking in order to be aware of negative habitual movements and search for alternative ones.

Two teachers, both suffering from back pain for many years, explained how the treatment made them aware of how they straightened their back and stopped breathing during daily activities and in particularly demanding situations. This enhanced awareness improved their understanding of their bodily complaints. One of them put it like this:

The treatment benefits me. I have learned a lot about myself. Now I can sense how I am straightening myself up too much, pulling myself together. I was not aware of that before.

Another patient, who experienced improved health, described the treatment as an educational experience:

The awareness process during the treatment has been like taking further education. For years, I have ignored my pain. I am now more aware factors increasing the pain, and my neck feels much better.

Despite a serious neck injury, she had continued to assume a lot of responsibility, not sparing herself. The treatment made her aware that ignorance of pain actually increased it. Attending to her own needs, and avoiding things like heavy lifting, had been recurrent topics during treatment.

The patients also described how the NPMP treatment process enhanced their awareness of the mutual connection between muscle tension and feelings.

One patient, with conflicts in her family, described enhanced awareness of how she clenched her teeth and how her heart rate increased when she got angry with her brother, who did not contribute in taking care of an ailing father. During treatment, she worked on finding other ways of expressing her anger, for instance, by walking it off and relaxing her jaw, rather than repressing it.

Most of the patients, except one, described how they, along with enhancing body awareness, improved their way of moving and functioning during daily routines. The experiences of less pain did also gradually change their attitude and relations towards themselves and their complaints. They searched for new ways of moving and acting and started changing some of their habits.

One patient, physically disabled because of the long-term neck and shoulder pain, described how she used to contact a chiropractor for emergency help, although her neck pain kept returning. Through NPMP treatment, in order to improve and sustain the improvement, she discovered that she needed to change her own bad habits of tensing up. She also realized that she was the only one to change this habit and explained:

When I started treatment, I was almost unable to dress myself. Now I am much better, although my change of work helped, too. I have gained an entirely different attitude towards what to do to improve my situation. I have learned that I am the one who has to do the work, and make changes.

Another patient, who endured widespread pain for many years, described how she after a few treatments became aware of how stress at work increased her bodily complaints. However, she did not notice her pattern of tension.

I remember, once stretching out on the bench after treatment I experienced an inner warmth, because I was able to relax.

She also noticed that her shoulders and jaw tightened up again when she hurried back to work after the treatment session. This enhanced awareness made her decide to give herself more time to rest after treatment, rather than immediately rushing back to work. Throughout her continued treatment, she became more aware of when her pattern of tension occurred, and she learnt some movements and strategies designed to counter this.

The middle-aged teacher who did not share these experiences had already made an effort changing some of her pattern of tension and was therefore convinced that surgery was the only solution to relieve her painful shoulder.

### To give oneself more space in daily life

In different ways, the patients gradually transferred their experiences from treatment into daily life. In general, this was a matter of giving themselves more space. However, in situations with demands from family members or colleagues, they were ambivalent about how much consideration they should have for themselves and how much for others. They tried to set aside moments in their daily routines to make space for enhancing awareness of and changing inefficient patterns of movement and actions. Several patients claimed that they should have made these efforts of change earlier in life. One patient offered insight through an expression of concern at the end of the interview:

I should have taken myself more seriously, earlier.

The patients described how enhanced awareness about tension and patterns of action helped them care more for themselves. They also described how this awareness gave them the opportunity for choice. One explained:

I practice saying yes to myself more often. This help me giving myself more space.

Furthermore, she added that she allowed herself pauses to get in touch with her own feelings by paying attention to them.

Several patients described how they worked on slowing down tempo. One patient shared how she worked on this, searching for more peace and quietness in life. In contrast, her partner preferred a fast pace, to be efficient in everything that needed to be done. She made a conscious effort to maintain her own preferred daily tempo. She experienced these minor adjustments in daily life to be rewarding since she immediately experienced less tension.

The patients described how they worked on changing their patterns of action and movement. One explained how she stopped being the person who always made coffee and tidied up the staffroom at work. She also practised changing her ways to act in interpersonal relations. Rather than withdrawing when she perceived colleagues to be difficult to deal with, she tried to be more open and express her opinions. Another patient explained how she practised not always offering help. Taking care of herself and avoiding exacerbated pain, she contributed in other ways than previously, either by asking for help to move furniture after a choir rehearsal or by doing less physically demanding tasks and exercises.

The patients chose different strategies to reduce tension and change dysfunctional movement patterns. One woman had devised a reminder—an hourly “ding” on her mobile telephone, which prompted her to stop.

When I hear the “ding”, I ask, “What is the posture of my shoulders now? How is my breathing?” Frequently, I find my shoulders raised a little, and my breathing being shallow. I then give myself space to release my shoulder and breathe.

Other patients explained how they linked new ways of moving to established daily routines. This might include, while brushing teeth or standing at a bus stop, to be aware of the contact of their feet with the ground, or lowering their shoulders and giving space to breathe while walking, or regulating the rhythm of movement when doing practical tasks. Some adopted a few exercises designed to calm themselves down, thus enhancing and transferring what they had learnt during the NPMP treatment into daily activities.

Most of the patients continued working on being more attentive and present in their daily lives. This might be, when they experienced the need for it, to give themselves pauses and space to relax, or performing movements, like releasing tense shoulders or stretching the neck. Exercises requiring materials and equipment were more readily excluded. At the same time, several patients replaced hard-core physical training with yoga and Pilates. One said:

I would never have thought that I should start Pilates exercise. I have always thought that it was not real exercise. Now I experience that it is good for me, and have decided to continue.

Ending treatment, most of the patients said that they felt well equipped and with improved health. Several of them further explained that now it was up to them, how to further apply what they had experienced and learnt, into their daily life.

## Discussion

The purpose of the present study was to explore experiences of change from NPMP treatment and transference of change into daily life situations. During the focus-group interviews, patients with long-lasting musculoskeletal pain shared their selected experiences. In the following discussion, the perspective of phenomenology of the body and previous research will provide a deeper understanding of the findings. Finally, we considered the methodological aspects of the study.

### Developing new relations towards oneself during treatment

At the start of treatment, the patients described how they had been living busy lives with many burdens and activities. Gradually, however, perceived pain restricted their activities and they were in need of treatment. According to Leder (1990), the body is taken for granted when it functions well. The body then slips away from attention, a kind of “disappearance”. However, when disability strikes, the body becomes the centre of attention as an obstacle disrupting habitual functions of daily life. The patients in the present study seemed to understand their symptoms differently at the start of the treatment, i.e.,, symptoms within a biomechanical frame or related to their lived lives of daily strain. Within a biomechanical frame, the body was understood as an object for repair. Related to daily strain, the body was ambiguous: both a subject experiencing the influence of lived lives and daily strain and a detached object to be observed. However, gradually over time, the patients described that the treatment made them more in touch with their bodies, reactions, feelings, and needs. The NPMP treatment is process oriented, and during the sessions, the patients are encouraged to be attentive to bodily sensations both during the “hands-on”, the movements and the verbal interaction (Thornquist & Bunkan, 1991). In the present study, the patients’ processes of change from perceiving the body in a restricted way at the start of treatment to enhancing embodied self-perception and awareness seemed to influence and change their understanding of the connection between symptoms, personal stories, and lifestyles. The two teachers experiencing how their back pain was linked to their habitually way of act and interact in concrete situations are examples. Such process of enhanced self-perception and awareness might be seen as a growing sensation of both being and having a body. Merleau-Ponty (2012) claims that we are our body and we perceive the world with our body. This improved self-perception also seemed to change their attitude towards themselves. According to Merleau-Ponty (2012), knowledge takes place within the horizons opened up by perception. To move from a pre-reflective to a reflective level brings meaning into existence and opens a new field of experience (Merleau-Ponty, 2012). The embodied self-perception and awareness the patients in the present study described also seemed to include a sense of embodied ownership, which revealed possibilities to explore new ways of using their bodies. This perception and awareness correspond to an immediate self-consciousness that involves senses of ownership and agency, as described by Gallagher (2000). Aided by improved perception of poor balance, restricted breathing, and muscle tension, the patients in the present study explored how to use less muscle power, to allow gravity to support their body during rest and movements, and to breathe more freely, both in the treatment sessions and in their daily lives. They explored alternative ways to be in positions like sitting and standing and in movements like walking in order to use less energy. Enhanced body awareness is a key construct in NPMP treatment, as well as a treatment outcome described in other studies of the treatment approach (Dragesund & Raheim, 2008; Ekerholt & Bergland, 2006, 2008; Øien et al., 2007, 2009; Sviland et al., 2012).

Whether the patients established new habits seemed to depend on their perception and understanding of bodily symptoms as well as the experience of becoming more in touch with themselves as bodily subjects. The experience of pain as contextual pain, like the patient clenching her teeth because of suppressed anger with her brother, facilitated a different way of acting. During the further treatment, one topic was to explore how to express anger in other ways, like relaxing her jaw and breath.

### Developing new habits in daily life

The process of becoming more in touch with themselves included being present in treatment situations by directing their attention towards their own body and needs. This, in turn, made up the basic elements of transference of experiences from the treatment into daily life, which mainly appeared as a matter of giving more space to themselves. Merleau-Ponty (2012) argued that freedom rests on being present in the situation. Thus, the possibility to establish new habits depends on the power of putting oneself into the situation. According to Stern (2006), body aspects like ownership of one’s body and movements influence the perception of self. The patients’ experiences of enhanced embodied self-perception, through the NPMP treatment, seemed to involve a new or different sense of ownership and agency. In addition, they involved themselves more in the process of change. According to Gallagher (2000), the sense of agency and the sense of ownership are pre-reflective, coincide, and indistinguishable. Further, Gallagher and Zahavi (2012) claim that a movement is an action if it is goal-directed and intentional, and an action is intentional if I sense that I decide to act for a reason. In line with this, the patients in the present study seemed to involve themselves more actively. They did take better care of themselves by relating more actively to their own bodily needs, possibilities, and limitations in different contexts. The described changes are in line with findings in the study by Øien et al. (2009). In their study, the patients’ basis for transferring experiences from treatment into daily life was linked to an enhanced awareness of own needs.

In the present study, the patients actively searched for new ways of moving, acting, and expressing themselves during their daily lives. In specific situations, they practised new acquired skills. A reminder with a “ding” on the mobile telephone, linking new movements to existing habits, and doing exercises designed to calm down and break their stress pattern were such examples. Other examples were changing habits in interpersonal relationships, like not always being the one offering help or take too much responsibility in concrete situations. Merleau-Ponty (2012) claimed that bodily based consciousness is a matter of I can or the general power of putting oneself into the situation. As if influenced by this, “matter of I can”, the patients created more space for their action during their daily lives and continued further to establish and sustain new habits. This type of experiences corresponds to findings from a focus-group study of NPMP therapists (Dragesund & Øien, 2018) who described how they by being bodily anchored and in touch with themselves enhanced self-agency and space for action during demanding treatment sessions.

Learning new skills and tasks, attention towards the moving body might be necessary. According to Gallagher (2000), body schema is a close-to-automatic system of the processes that constantly regulate posture and movement during intentional action. It reflects the body’s adjustment to the environment, without the patient thinking about how the body copes with the difficulties. Thus, the body schema is connected to the senses and the adaptation between the sensory system and the motor system attuning the body to the physical-, social-, and cultural environment. In contrast to the body schema, the body image consists of a system of perceptions, attitudes, and beliefs pertaining to one’s own body (Gallagher & Zahavi, 2012). The patients’ new way of moving might have expanded the body schema which, in turn, might have influenced the body image. Their experience of being more in touch with their own body opened up the possibility to be aware of and reflect upon earlier movements and actions and to explore how to change these in daily life contexts. The changes the patients transferred into daily life context were all adjustments of movements and actions they perceived released their muscle tension, rhythm of breathing, or movements. They gradually experienced new movements (habits) being developed alongside prior ones.

### Methodological considerations

The included patients might have skewed the sample, in which most of them had positive experiences from the NPMP treatment. However, the discourse within each focus group was not the focus of the analysis. Our interest was to grasp the individual patients’ experiences from the treatment, searching for common themes.

Validity, reflexivity, and relevance influence the findings in qualitative research (Malterud, 2001b). The fact that both researchers are experienced NPMP therapists, and that the first author is still working as a clinician, might have influenced the findings but could also have added strength to the validity of the findings. Reflexivity was handled through discussions and negotiations at all stages of the research process. The content of the results is limited to the participants in the study. However, the results might be relevant to comparable therapeutic settings, depending on whether the knowledge is experienced as meaningful and trustworthy. The value of the results should also be evaluated according to relevance and practical use (Malterud, 2001b). An analytical generalization, however, is possible when empirical results are discussed with previously developed theory (Yin, 2003). In the present study, the results are discussed with the phenomenological perspectives of the body.

### Implications for physiotherapy practice

The study indicates that how patients define their bodily symptoms has an impact on self-perception. Then, in turn, enhanced embodied self-perception, ownership, and agency seem to influence both the awareness of own bodily needs and transferring changes from treatment into daily life. Thus, knowledge about how patients perceive and understand their pain and bodily symptoms may improve the understanding of factors preventing or facilitating the process of change during treatment. The study further indicates that if patients should maintain new habits, it is of importance to further work on being present and in touch with own movements and pattern of action during daily life. Physiotherapy might be useful in guiding patients how to transfer experiences from treatment into daily life context.

## References

[cit0001] Braatøy, T. (1947). *De nervøse sinn* [The nervous mind]. Oslo: Cappelen.

[cit0002] Dragesund, T., & Øien, A. M. (2018). Demanding treatment processes in Norwegian Psychomotor Physiotherapy: From the physiotherapists’ perspectives. *Physiotherapy and Practice*, 35(3), 1–9. 10.1080/09593985.2018.1463327 29683774

[cit0003] Dragesund, T., & Raheim, M. (2008). Norwegian Psychomotor Physiotherapy and patients with chronic pain: Patients’ perspective on body awareness. *Physiotherapy Theory and Practice*, 24(4), 243–254. 18574750 10.1080/09593980701738400

[cit0004] Ekerholt, K., & Bergland, A. (2008). Breathing: A sign of life and a unique area for reflection and action. *Physical Therapy*, 88(7), 832–840. 18483131 10.2522/ptj.20070316

[cit0005] Ekerholt, K. B., & Bergland, A. (2006). Massage as integration and a source of information. *Advances in Physiotherapy*, 8(7), 832–840.

[cit0006] Gallagher, S., II. (2000). Philosophical conceptions of the self: Implications for cognitive science. *Trends in Cognitive Sciences*, 4(1), 14–21. 10637618 10.1016/s1364-6613(99)01417-5

[cit0007] Gallagher, S. Z., & Zahavi, D. (2012). *The phenomenological mind* (2nd ed.). London: Routledge.

[cit0008] Giorgi, A. (2009). *The descriptive phenomenological method in psychology: A modified husserlian approach*. Pittsburg, PA: Duquesne University Press.

[cit0009] Krueger, R. A., & Casey, M. A. (2000). *Focus groups. A practical guide for applied research* (3rd ed.). Thousand Oaks, London: Sage Publications.

[cit0010] Kvåle, A., Ljunggren, A. E., & Johnsen, T. B. (2003a). Examination of movement in patients with long-lasting musculoskeletal pain: Reliability and validity. *Physiotherapy Research International*, 1(8), 36–52. 10.1002/pri.270 12701464

[cit0011] Kvale, S. B., & Interviews, S. (2009). *Learning the craft of qualitative interviewing*. Los Angeles: Sage.

[cit0012] Leder, D. (1990). *The absent body*. Chicago: The University of Chicago Press.

[cit0013] Malterud, K. (2000). Symptoms as a source of medical knowledge: Understanding medically unexplained disorders in women. *Family Medicine*, 32(9), 603–611. 11039146

[cit0014] Malterud, K. (2001b). Qualitative research: Standards, challenges, and guidelines. *The Lancet*, 358(9280), 483–488. 10.1016/S0140-6736(01)05627-6 11513933

[cit0015] Merleau-Ponty, M. (2012). *Phenomenology of perception*. nd, editor. London: Routledge.

[cit0016] Morgan, D. L. (1997). *Focus groups as qualitative research*. Thousand Oaks, CA: Sage Publications.

[cit0017] Øien, A. M., Iversen, S., & Stensland, P. (2007). Narratives of embodied experiences—Therapy processes in Norwegian Psychomotor Physiotherapy. *Advances in Physiotherapy*, 1(9), 31–39.

[cit0018] Øien, A. M., Råheim, M., Iversen, S., & Steihaug, S. (2009). Self perception as embodied knowledge-changing processes for patients with chronic pain. *Advances in Physiotherapy*, 11(3), 121–129.

[cit0019] Polit, D. F. (2017). *Nursing research: Generating and assessing evidence for nursing practice* (10th ed.). Philadelphia: Lippincott Williams og Wilkins.

[cit0020] Steihaug, S. (2005). Can chronic pain be understood? *Scandinavian Journal of Public Health*, 66, 36–40. 16214721 10.1080/14034950510033354

[cit0021] Stern, D. N. (2006). *The interpersonal world of the infant: A view from psychoanalysis and development psychology*. London: Karnac.

[cit0022] Sviland, R., Raheim, M., & Martinsen, K. (2012). Touched in sensation–moved by respiration: Embodied narrative identity—A treatment process. *Scandinavian Journal of Caring Sciences*, 26(4), 811–819. 22716182 10.1111/j.1471-6712.2012.01024.x

[cit0023] Thornquist, E., & Bunkan, B. H. (1991). *What is psychomotor physiotherapy*. Oslo: Norwegian University Press.

[cit0024] van Manen, M. (2014). *Phenomenology of practice: Meaning-giving methods in phenomenological research and writing*. London: Routledge.

[cit0025] Yin, R. K. (2003). *Case research. Design and methods*. Thousand Oaks, CA: Sage Publications.

[cit0026] Zahavi, D. (2018). Getting it quite wrong: Van Manen and Smith on phenomenology. *Qualitative Health Research*, 19, 1–8. 10.1177/1049732318817547 30565516

